# For-Profit Hospitals Have Thrived Because of Generous Public Reimbursement Schemes, Not Greater Efficiency: A Multi-Country Case Study

**DOI:** 10.1177/0020731420966976

**Published:** 2020-10-27

**Authors:** Patrick P. T. Jeurissen, Florien M. Kruse, Reinhard Busse, David U. Himmelstein, Elias Mossialos, Steffie Woolhandler

**Affiliations:** 1 IQ Healthcare Scientific Institute for Quality of Healthcare, Radboud Institute for Health Sciences, Radboud University Medical Center, Nijmegen, the Netherlands; 2 Ministry of Health, Welfare and Sport, The Hague, the Netherlands; 3 Department of Health Care Management, Berlin University of Technology, Berlin, Germany; 4 City University of New York at Hunter College, New York, New York, USA; 5 Harvard Medical School, Cambridge, Massachusetts, USA; 6 Department of Health Policy, London School of Economics and Political Sciences, London, UK

**Keywords:** for-profit hospitals, health policy, delivery of health care, private sector, comparative study, organization and administration

## Abstract

For-profit hospitals’ market share has increased in many nations over recent decades. Previous studies suggest that their growth is not attributable to superior performance on access, quality of care, or efficiency. We analyzed other factors that we hypothesized may contribute to the increasing role of for-profit hospitals. We studied the historical development of the for-profit hospital sector across 4 nations with contrasting trends in for-profit hospital market share: the United States, the United Kingdom, Germany, and the Netherlands. We focused on 3 factors that we believed might help explain why the role of for-profits grew in some nations but not in others: (1) the treatment of for-profits by public reimbursement plans, (2) physicians’ financial interests, and (3) the effect of the political environment. We conclude that access to subsidies and reimbursement under favorable terms from public health care payors is an important factor in the rise of for-profit hospitals. Arrangements that aligned financial incentives of physicians with the interests of for-profit hospitals were important in stimulating for-profit growth in an earlier era, but they play little role at present. Remarkably, the environment for for-profit ownership seems to have been largely immune to political shifts.

In recent decades, for-profit hospitals have gained market share throughout many developed nations. Conventional wisdom may attribute for-profit hospital success to greater efficiency.^1–3^ However, we argue that this claim is based on unfounded assumptions, and we analyze the growth and development of the for-profit hospital sector in 4 countries in order to explore alternative explanations.

## Hospital Ownership: Public, Nonprofit, and For-Profit

Many nations’ health systems include a variety of hospital ownership types: for-profit, nonprofit, and public. Public hospitals are legally part of the government, either as state-owned organizations at arm’s length or fully owned by regional or local governments.^4^ Nonprofit hospitals must use any surpluses (or profits) to further their organizational purposes or missions, and they are barred from distributing surpluses to individuals who exercise control over them.^5^ Conversely, for-profit hospital owners control their organizations and have the right to all “residual claims” (i.e., the profits) after all prior obligations have been paid.^6^

The for-profit hospital sector comes in many shapes and sizes, ranging from small, physician-owned institutions to large, publicly traded for-profit hospital chains. Increasingly, small, individual for-profit hospitals are being consolidated into (very) large investor-owned chains.^7^ Depending on country context and regulation, for-profits often specialize in lucrative areas of care, such as elective surgery,^8^ and are more likely to target private-pay (or privately insured) patients.^9^

Kenneth Arrow,^10^ a founding father of health economics, argued that fundamental information asymmetries in health care markets mandate reliance on trustworthy agents to compensate for market failures. He suggested that for-profit organizations cannot satisfy this standard because “[t]he very word, ‘profit,’ is a signal that denies the trust relations” (p.965).^10^ Following this line of thought, one may believe that nonprofits, with a status signaling that their objectives are not to maximize profits, might therefore be best suited to act in the interest of patients. However, in another health economics classic, Pauly and Redisch^11^ postulate that shrinkages in the U.S. proprietary hospital sector reflect powerful physician interests, because nonprofit hospitals operate as *de facto* doctors’ facilities and are effectively for-profits in disguise, whereby physicians exercise authority over hospital assets in order to maximize their income without running financial risks.

Both Arrow’s^10^ and Pauly and Redisch’s^11^ analyses suggest that nonprofits would dominate the hospital sector. However, several countries on different continents have seen an expansion in the for-profit hospital market in recent years.^12–15^ This growth in for-profit share of the hospital sector raises puzzling questions.

## Why Is It That For-Profit Hospitals Do Not Deliver Superior Performance?

Many economists hold that for-profit ownership is naturally more efficient because in theory, these institutions must continuously strive to outperform nonprofit or public organizations in order to maximize profit and satisfy their shareholders.^11,16–18^ However, empirical evidence contradicts this. Systematic reviews analyzing the relationship between hospital ownership and quality of care have either found mixed results^9,19,20^ or have favored nonprofit or public providers.^21,22^ Reviews of hospital efficiency have arrived at the same conclusion: There are mixed results, but generally, for-profit providers do not outperform other ownership types.^9,21,23^For-profit hospitals tend to charge higher prices than public and nonprofit hospitals.^19,23,24^ This, in part, may reflect their wider profit margins^25,26^ and higher overhead and capital expenditures.^27–30^ Despite higher costs to the payor, for-profit hospitals often outsource and are thus able to minimize the number of employed staff – particularly non-physician staff.^31^ As a result, for-profit hospitals typically benefit from lower personnel costs.

Interpreting empirical findings on this topic requires the consideration of 3 important nuances. First, many systematic reviews on this subject have highlighted the complexities around drawing conclusions^9,23,32–34^ when there is substantial variability within different ownership types.^20^ Second, exogenous economic incentives might at times override provider missions and goals. For instance, spillover effects can impact and alter the motives of nonprofit organizations. Such spillovers might be beneficial or detrimental. For example, for-profit providers’ entry in the market might push nonprofits to adopt similar structures and strategies.^35,36^ Nonprofit hospitals may feel pressured to increase their efficiency or to focus on profitable services such as elective surgeries and minimize charity care.^25,37–41^ Third, while some cross-sectional studies have found that for-profits are less efficient because they tend to acquire inefficient public and nonprofit organizations,^9^ other longitudinal studies suggest that for-profit entities streamline the public and nonprofit hospitals they acquire and thereby achieve greater efficiency.^26,33^

## Research Questions

If, as the literature suggests, consistently superior performance on patient outcomes or economic efficiency does not explain the growth of for-profit hospitals, other factors must be explored.

### How Does Access to Capital and Payment for Services Vary by Hospital Ownership Type?

All hospitals require access to capital funds for investments into new or upgraded facilities that are essential for growth and even survival; however, they depend upon different sources for these capital funds. For-profits can attract capital from investors who seek a share of the earnings (i.e., venture capital firms and the stock market) and can also raise funds through bank loans or by issuing bonds.^42^ Nonprofits can tap into philanthropic funds, receive government grants, issue (tax-exempt) bonds, and retain earnings from operating surpluses.^42,43^ On the whole, nonprofit organizations’ financing costs are lower.^39^ However, in some circumstances, for-profits have an advantage: For example, a for-profit hospital with a high stock-price-to-earnings ratio may yield more by raising capital through stock sales rather than by borrowing.^39,44,45^ In other words, the relative costs of different sources of capital fluctuate, and such fluctuation can turn the tables in defining which ownership type has a financing advantage. Furthermore, the growth of the for-profit sector may be hindered if government-regulated health financing plans limit or disfavor them.

### How Do Physician Incentives and Influence Vary Across Different Types of Hospital Ownership?

Physicians often exert considerable influence over hospital management^46^ and a hospital’s business prospects.^47^ While many factors shape physician working conditions and job satisfaction, remuneration certainly plays a role. For-profit entities may offer physicians higher pay (e.g., in the form of an ownership stake in the firm),^28^ but they may also reduce (non-)physician employee pay in order to maximize profits. This incentive structure is absent or may be weaker in nonprofit organizations. Employment in nonprofit organizations might be attractive to physicians because of commitments to social and altruistic goals.^5,48^ For some physicians who recognize that (as Pauly and Redisch noted) nonprofit hospitals can be for-profits in disguise, the attraction of a nonprofit hospital might be linked to physicians’ desires to maximize their incomes.^11^

### Does the Ruling Political Party Determine the Success of Different Hospital Ownership Types?

Political theory would predict that left-leaning government regimes are more likely to be anti-commercial and hence to implement public policies that disfavor for-profits. In contrast, theory predicts that right-leaning politicians are more apt to trust market forces in health care and to implement for-profit-friendly health policies.

## Structure of the Article

In this article, we examine the role of these 3 factors in the for-profit hospital market. We consider: (*a*) public policies granting access to capital and payments for services, (*b*) physicians’ stake in for-profit medical enterprises, and (*c*) the political milieu, and we compare these trends in for-profit market growth in 4 countries. Below, we outline the methods and data that inform our study; we then present an overview of the trends of for-profit market share across the 4 countries over time; following this, we delve deeper into our 4 case studies and demonstrate the similarities and differences across the for-profit hospital sector in these countries; finally, we discuss the lessons learned and policy implications of our findings and offer several conclusions.

## Methods and Case Selection

We conducted a historical case study of the growth and characteristics of the for-profit hospital sector and health care environment in 4 nations (Table 1). We included cases with substantial (Germany and the United States) as well as negligible (the Netherlands) for-profit sectors. Our cases cover the spectrum of health financing systems: mainly privately funded (United States), publicly funded (United Kingdom), and those funded by social insurance (Germany and the Netherlands). These 4 cases can also be stratified in such a way that they are relevant in answering our research questions. The United Kingdom and Germany both rely on public capital subsidies and regulation. These are centralized in the United Kingdom and decentralized in Germany. Hospital capital (and debt repayment) in both the United States and the Netherlands is largely funded by operating surpluses that hospitals generate internally from reimbursement fees paid by insurers for care provided. Hospital physicians are mainly paid salaries in both Germany and the United Kingdom. Until recently, these physicians in the United States were typically self-employed; in the Netherlands, about half of hospital physicians are self-employed and half are salaried.^49^ Political discussion on the appropriateness of for-profit hospitals has arisen in previous decades. It was prominent in the United Kingdom during the mid-1970s; in the United States during the 1980s (and again, regarding physician-owned specialty hospitals from the early 2000s onward); in Germany in the early 1990s; and in the Netherlands in the first 10 years after the 2006 health care market reform.

**Table 1.** Characteristics of the Health Systems and Size of the For-Profit Hospital Sector in the United States, United Kingdom, Germany, and the Netherlands.

	U.S.	U.K.	Germany	Netherlands
Number of for-profit hospitals(% of total) [Year]	1,645(26.6%)[2015]	195^a^(11.1%) [2014]	720(37.1%) [2017]	1^b^ (1.4%)[2018]
For-profit beds (% of total) [Year]	173,758(18.5%)[2015]	8,730^c^(5.0%)[2018]	93,189(18.7%)[2017]	257^b^ (0.7%)[2018]
Health system	Private with public programs	National Health Service	Social-insurance	Social-insurance
Capital funding	Mainly operating surpluses	Public subsidies	Mainly public subsidies	Mainly operating surpluses
Physician employment status	Mainly self-employed until recently, currently mixed	Salary / self-employed (private sector)	Salary	Self-employed / salary
Explicit political debate	Effects of profit making (1980s), cherry picking by specialty hospitals (2000s)	NHS pay-beds (1970s) and outsourcing to the private sector (1980s)	Privatization of hospitals in former German Democratic Republic (1990s)	Lifting ban on profit distribution (2008–2019)

Sources: AHA,^50^ LaingBuisson,^51^ OECD,^52^ Statistisches Bundesamt,^53^ and CIBG.^54^

^a^These figures reflect all non-NHS hospitals and exclude day facility-only private hospitals.

^b^Two hospitals owned by private investors that went bankrupt in 2018 are excluded from the table.

^c^These figures reflect all beds in the independent acute medical care hospitals.

We collected data on the for-profit hospital sectors in 4 nations using official statistics, secondary sources, gray literature, and peer-reviewed studies.

## Results

### For-Profit Hospital Market Share: Overview of Findings

**Figure 1.**
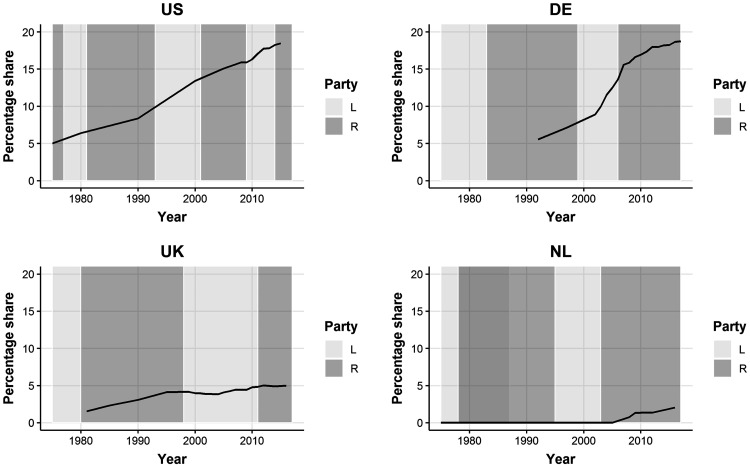
Trends in for-profit hospitals’ share of total beds in each nation, and the political leanings of the ruling party during each period.^a^ ^a^Authors' calculations. Figures reflect inpatient (acute care) beds. The Dutch figures reflect the acquisition of three hospitals by a commercial firm, but because missing data on acquisition dates, the graph may be imprecise.*Sources:* AHA (2017)^50^, CDC (2017)^55^, OECD (2019)^52^, OHE (2011)^56^, LaingBuisson (2017)^57^, LaingBuisson (2019)^51^, Statistisches Bundesamt (2018)^53^, CIBG (2020)^54^

Figure 1 displays trends in for-profit share of hospital beds in each nation and the political leanings of the governments over time. For-profit market share has grown rapidly in Germany and the United States, currently exceeding 15% in each of these nations. In the United Kingdom, growth has been more modest, and private hospital beds currently account for 5% of the total (U.K. figures are for all non-National Health Service [NHS] hospitals, including nonprofits such as Nuffield and London Clinic, which accounted for 12.9% of private hospital beds in 2018).^51^ In the Netherlands, only a single hospital remained under for-profit ownership following the 2018 bankruptcy of 2 hospitals that had been acquired by commercial investors.

Because for-profit hospitals are generally smaller than nonprofit and public hospitals, their market share as measured by the number of hospitals is higher than their share of beds: These shares are 26.7% in the United States in 2018 (up from 17.9% in 2000) and 35.8% in Germany (up from 21.7% in 2000) (authors’ calculations; no comparable data are available for the United Kingdom).^50,53^

Surprisingly, for-profit hospital growth rates in the United States and Germany appear largely unrelated to the political leanings of the governing party. For-profit growth in the United Kingdom coincided with the vogue for New Public Management (NPM) starting in the late 1980s. While it is difficult to define left- vs. right-leaning in the Dutch or German context because these governments are sometimes (i.e., Germany) or always (i.e., Netherlands) coalitions between parties, political milieu appeared to have little relation to for-profit hospital growth. The indicated political leanings of the Dutch government in Figure 1 are based on the largest party in the coalition in each period.

## The Early Roots of the For-Profit Hospital Sector

In the late 19th century, almshouses (United Kingdom), philanthropic institutions (United States), and religious providers (Netherlands and the United States) that had previously provided medical care to the destitute began to be replaced by modern hospitals with sophisticated operating theaters and diagnostic equipment that catered to patients of all economic backgrounds.^12^ Most of these early hospitals were publicly or church-owned facilities located in city centers. In bigger cities, many hospitals limited admitting privileges to a small group of physicians, which stimulated the growth of physician-owned clinics that tended to target wealthier patients. However, the financial prospects of the emerging for-profit hospital sector were lackluster. They could neither tap into low-cost charitable or public sources of capital nor could they use cheap religious labor such as nuns, and public payments for care of the poor were meager.

The 1930s depression dealt a major blow to the for-profit hospital sector in many nations.^12^ While data is limited, we know that in Germany, proprietary hospitals’ share of beds declined from 7.0% in 1931 to 5.9% in 1937.^58^ In the United Kingdom, 9.6% of all beds were in private nursing homes in 1921, declining to 7.2% in 1938.^59^ U.S. proprietary facilities accounted for 17.3% of hospital beds in 1928, but no more than 9.5% in 1940.^12^

Shortly after World War II, many Western countries developed or cemented their welfare states, increased public expenditures on health care, and, in several cases, implemented universal health coverage.^60^ However, in most nations, the expanded public financing of health care afforded only a marginal role to for-profit hospitals, casting a shadow over this sector. The eclipse of for-profit hospitals that prevailed at the time of Arrow’s and Pauly and Redisch’s^11^ analyses led them to conclude that nonprofits would remain dominant in the health care sector. With the benefit of hindsight, it seems these eminent scholars miscalculated.

### The United States

#### Medicare and Medicaid Capital Payment Policies

In the United States, the proprietary hospital sector bottomed out in the early 1960s, and its renewed growth coincided with the start-up of Medicare (1965) and Medicaid (1966). This was no coincidence: Both programs created huge financial opportunities for hospitals, particularly for for-profits.

Medicare, which covered persons age 65 and over, paid hospitals for their operating costs, with a 2% add-on for future “capital improvements” and additional payments for existing capital costs (such as interest on debts and depreciation).^61^ While the Hill-Burton program that provided massive federal grants for hospital construction starting in 1946 barred for-profit hospitals from participating,^62,63^ Medicare (and most state Medicaid programs, which cover some of the poor) offered for-profits extra payments that were unavailable to nonprofit or public facilities. This additional capital payment for return on investment was set at 1.5 times the rate of return earned by Medicare’s Hospital Insurance Trust Fund.^12^ This proviso, inserted at the insistence of the nursing home industry, virtually guaranteed for-profit facilities a “risk-free” investment return.^61^

Medicare’s and Medicaid’s capital payment policies spurred the rapid growth of hospital firms such as HCA (previously Hospital Corporation of America), which was founded in 1960 and by 1980 owned about 300 hospitals with 40,000 beds. Much of that growth came from acquisitions that were effectively subsidized by the public program, which (in addition to the generous payments discussed above) reimbursed for-profits for their interest payments on debts incurred to purchase additional hospitals.^64,65^ Moreover, tax laws permitted owners of hospital buildings to claim accelerated depreciation over a 15-year period. These measures assured for-profit hospitals of ready and cheap access to funds for new investments. By the early 1980s, for-profit providers were receiving 40% of all capital reimbursements nationally, although they accounted for only 7.6% of total hospital expenses.^64^ This favorable public reimbursement plan stimulated the creation of new hospitals and the consolidation of the for-profit sector (Table 2).

**Table 2.** Growth of Investor-Owned Hospital Chains Around 1980 in the United States.

	Number of chain-owned hospitals	Percentage of total hospital beds	Number of stand-alone for-profit hospitals
1975	378	5.2%	682
1980	531	7.5%	NA
1982	682	8.9 %	330

Source: Gray.^64^

#### Market-Driven Health Care Reforms During the Reagan Administration

The Reagan Administration’s (1981–1989) health policies were driven by its stated desire to reduce government spending and introduce market-based principles, an approach resembling the NPM ideology ascendant around the same time in the United Kingdom.

In 1982, the average profit margin of for-profit hospital chains was more than double that of the hospital sector as a whole, 9.2% versus 4.3%.^66^ While advocates saw this as an indication of more effective management,^62^ the growth of investor-owned hospital chains provoked increasing debate, leading the Institute of Medicine to undertake the first large-scale study of for-profit hospitals in 1986.^64^ The Institute panel concluded, ambiguously, that for-profit ownership was having an important effect on the health system, but that the available evidence was insufficient to justify policies either opposing or supporting investor ownership.^64^

The administration’s political bent precluded taking any steps that directly challenged the existence of the for-profit hospital sector. However, starting in 1982, the generous capital reimbursements to for-profit providers were gradually phased out after the publication of highly critical reports by the U.S. General Accounting Office.^67^ The return-on-equity payment rate was cut from 1.5 to 1.0 times the rate of return of the Hospital Insurance Trust, and the option to charge Medicare for acquisition costs was discontinued by the Deficit Reduction Act of 1984.^68^

In 1983, Medicare replaced cost-plus hospital reimbursement with a system based on diagnostic-related-groups (DRGs).^69^ DRG proponents hoped the shift would stimulate efficiency and moderate hospital costs. Initially, the for-profit sector welcomed the new payment approach, anticipating that it would reward more efficient providers and hence be to its advantage. But things turned out differently. Reports of high hospital profit margins led Congress to repeatedly reduce annual payment rate increases, which cut profits.^70^ Capital costs and return on equity payments were gradually folded into DRG payments, rather than being add-ons, as under Medicare’s prior payment system. By 1992, for-profit hospitals were no longer receiving the extra payments they had enjoyed since 1966. Moreover, adverse publicity generated by the practice of patient dumping of critically ill uninsured patients^71^ triggered passage of the 1986 Emergency Medical Treatment and Labor Act, which to this day requires emergency departments to stabilize urgently ill patients regardless of ability to pay,^72^ crimping for-profits’ ability to avoid unprofitable patients.

The for-profit hospital industry’s exuberant expenditures on lobbying indicate the importance it has placed on political and regulatory decision making. In 1985, the industry accounted for 36% of all hospital lobbying expenses and 30% of hospitals’ contributions to political candidates, while its trade association funded another 25% of contributions.^73^ Despite these contributions, for-profits encountered some new policy constraints, but kept on growing.

#### The Managed Care Era

Starting in the 1980s, traditional health insurance that paid virtually anything that any provider charged gradually gave way to managed care plans, which negotiated lower prices, imposed strict utilization management, and restricted provider networks.^74^ The price reductions, narrow networks, and utilization reviews reduced hospital utilization and left hospitals with excess capacity.^75^ The financial pressure on hospitals was intensified by the 1997 Balanced Budget Act, which initiated 3 years of meager Medicare payment rate increases. For-profit hospitals’ revenues stalled, and the acquisition value per bed was halved.^76^

For-profit hospital chains responded by reshaping themselves into locally dominant systems (i.e., oligopolies) with the muscle to extract higher prices from private payers. They also initiated grassroots (or “Astroturf”) campaigns to loosen the restraints imposed by the Balanced Budget Act and contributed to the managed care backlash of the late 1990s; this pushed many private payers to shift to plans (such as preferred provider organizations) that had less restrictive networks (although they also typically came with higher copayments).^77^

Several other strategies have bolstered the for-profit hospital sector’s resilience in the United States, despite less favorable reimbursement regulations and increasing penetration of managed care. For-profits have diversified through activities such as psychiatric inpatient care and have applied rigorous “turnaround management” to failing public and nonprofit hospitals they have acquired. Some firms have reaped profits by acquiring cash-strapped hospitals sitting on valuable real estate and selling off the buildings. For-profit hospitals have also sometimes profited by manipulating complex rules: for example, purchasing publicly financed assets at below-market prices. Finally, several of the largest for-profit firms have engaged in outright fraud and abuse, including large-scale up-coding (portraying patients as sicker than they really are to maximize reimbursement).^78^ HCA, still the largest for-profit chain, paid $840 million to settle charges of engaging in such inappropriate practices,^79^ while another for-profit hospital organization, Tenet, has paid millions in fines for overbilling Medicare for cardiac surgery.^80,81^

#### Physician Incentives and Participation in the For-Profit Sector

Although an increasing proportion of U.S. physicians are employed by hospitals,^82^ historically, most have been self-employed and affiliated with one or more hospitals. In earlier decades, for-profit hospitals offered physicians financial incentives, such as an equity stake in a local venture, to admit patients.^12^ Starting in the 1980s, for-profit and other general hospitals faced increasing competition for lucrative patients from outpatient surgery centers and physician-owned specialty hospitals offering a limited range of services, such as orthopedic and cardiac surgery.

Specialty hospitals were particularly threatening for the existing general for-profit hospital industry because of their focus on high-revenue services and the rapid growth in their patient volumes. In December 2003, Congress imposed an 18-month moratorium banning new physician-owned specialty hospitals from billing Medicare. While the American Medical Association had, until 1984, discouraged physician ties to for-profit hospitals, in 2004 it opposed extending the moratorium – opposition that was overridden by hospital groups that lobbied intensively against specialty hospitals’ “unfair” competition. In 2005, Congress re-imposed the moratorium^83^; however, it was lifted again in 2006.^84^

At present, wages for non-physician hospital employees are generally lower at for-profit than at nonprofit hospitals, a reversal of the pattern in 1990.^85^In contrast, for-profits often offer physicians lucrative arrangements in the form of incentive-based payments^86^ or a share of hospital profits.^87^

#### Recent Developments: The Affordable Care Act, the Trump Administration, and the COVID-19 Crisis

The most important effect of the 2010 Affordable Care Act (ACA) was a reduction in the uninsured rate from 15.5% in 2010 to 8.6% in 2016.^88^ The decline of the number of uninsured benefited the for-profit sector by reducing bad debt and free care, although this has been offset by rising copayments that have led to increases in bad debts among persons with coverage.^89^ The ACA also implemented accountable care organizations (ACOs) and so-called value-based purchasing programs in Medicare, which have had mixed effects on hospital margins. Moreover, the vast majority of hospitals participating in ACOs are nonprofits.^90^

In addition, the ACA cut the annual increase in Medicare’s payment rates for hospitals, widening the gap between the rates paid by public versus private insurers^91^ and increasing the incentives to recruit privately insured patients.^92^ Of particular relevance to for-profit hospitals, Section 6001 of the ACA placed new restrictions on existing physician-owned specialty hospitals and reinstated a moratorium on payments to new ones. While several such hospitals rushed to open before the moratorium came into effect, their numbers subsequently fell, to the advantage of other for-profit hospitals.^93^

**Figure 2.**
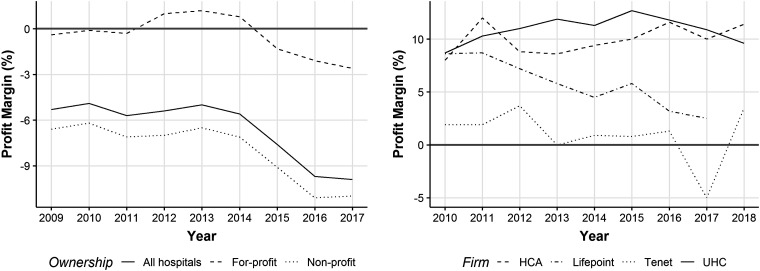
Trends in Medicare margins of all US hospitals (left panel) and the profit margins of the largest for-profit hospital firms (right panel).^a^ ^a^Margins in the left graph are calculated as payments minus Medicare-allowable costs, divided by payments. “Overall Medicare margin is for acute inpatient, outpatient, hospital-based skilled nursing facility (including swing beds), hospital-based home health, and inpatient psychiatric and rehabilitation services, plus uncompensated care, graduate medical education, and electronic health record incentive payments” (p.85)^94^ The margins in the right graph are calculated as Earnings Before Interest, Taxes, Depreciation and Amortization (EBITDA).*Sources:* MedPAC (2017)^94^, MedPAC (2019)^95^, Bureau van Dijk (2020)^96^, HCA Healthcare (2015;2013;2010)^97,98,99^, Universal Health Services (2018;2015;2013;2010)^100,101,102,103^, Tenet Healthcare Corporation (2018; 2015;2013;2010)^104,105,106,107^

On the whole, it appears that nonprofit and public hospitals have borne the brunt of adverse financial consequences from the ACA, while for-profits have continued to prosper, as illustrated by their more favorable Medicare margins (Figure 2) and by the fact that the profit margins of the largest for-profit chains have remained relatively stable or increased.

Until the COVID-19 outbreak, for-profit hospitals have fared particularly well during the Trump administration. While the corporate tax cuts enacted in 2017 attenuated the tax exemption advantage of nonprofit hospitals,^108^ they saved the largest for-profit chains an estimated $800 million in 2018.^109^ And since Trump assumed office, Medicare reimbursement rates have increased, benefiting both nonprofit and for-profit hospitals.^110^

Most recently, the COVID-19 pandemic has damaged the finances of for-profit hospitals^111^ because, as one firm said in a statement, “Elective surgeries are the cornerstone of our hospital system’s operating model – and the negative impact due to the cancellation of these procedures cannot be overstated.”^112^ At the time of this writing, the long-term repercussions of the pandemic on for-profit hospitals remain uncertain.

### The United Kingdom

#### For-Profit Hospitals in a Country With a National Health Service

The NHS, established in 1948, promised care “free at the point of delivery” to all. The Labour Government nationalized almost the entire hospital sector. Only some nonprofit hospitals remained outside the NHS at the time, and several private insurers, anticipating that demand for private insurance would persist, formed the British United Provident Association (BUPA), which, in 1949, covered 34,000 subscribers.^12^ Until the 1970s, this so-called independent sector had modest growth. While hospitals outside the NHS originally comprised primarily nonprofits, this independent sector transitioned to mostly for-profit ownership over time.

To enlist senior specialists’ (consultants’) crucial support for the NHS,^113^ the government allowed them to engage in some lucrative private practice within NHS hospitals, using so-called pay beds. Pay-bed payment rates were very high, although the number of patients who used them was small.^114^ Nevertheless, these pay beds were very important for the income of consultants, and the NHS’s founding father, Aneurin Bevan, famously described: “I stuffed their mouths full with gold”.^115^

Initially, pay-bed care was mainly financed through out-of-pocket payments. While the role of private insurance grew over time,^116^ by as late as 1975, 40% of bills for private care in the NHS were still paid out-of-pocket.^117^ When some nonprofit hospitals began to be incorporated into the NHS, the availability of private care was limited and private insurers were increasingly anxious to expand the supply of private providers for their clients. In 1957, BUPA, by far the largest private insurance company, donated a substantial sum to facilitate the emergence of the first private nonprofit hospital chain, known as the Nuffield Hospitals. By 1967, Nuffield was operating 13 hospitals, which grew to 26 in 1976.^118^ It remains a nonprofit, but commercially influenced private hospital chain.

#### Commercial Conversions in the For-Profit Sector

During the 1970s, private hospital care triggered heated debate. In 1974, the Labour Government, supported by the unions, tried to simultaneously limit the number of NHS pay beds and severely curtail the independent sector.^119^ They harvested the opposite: a much more commercial independent hospital sector. The government’s policies posed a direct threat to the income of NHS consultants who pursued private practice. Many consultants were outraged, and massive strikes loomed. A coalition of private insurers and private hospitals managed to gather the support of the British Medical Association (BMA) to block implementation of these policies.^120^ The government compromised: The number of pay beds would be reduced, but less than had been previously planned, and the government promised less interference with the independent sector. However, an unintended consequence was that NHS consultants began to refer large numbers of their private patients to the independent sector.

Spurred by new opportunities, the independent hospital sector took on an increasingly for-profit character, as new for-profit providers stepped into the market. BUPA founded its own for-profit hospital subsidiary. U.S. hospital chains opted to enter the United Kingdom, which served as a pilot to test whether they could find success outside their home country. These groups invested heavily in new facilities and equipment.

The prospects of the young for-profit hospital sector greatly improved after Margaret Thatcher’s rise to prime minister in 1979 and the ascendancy of NPM ideology in the NHS, which was fueled by the Griffiths report.^121^ Retrenchment of the public sector was at the core of this ideological project. NHS budgets were curtailed, causing large increases in waiting lists for elective surgery and making private alternatives more attractive. The government also encouraged public purchasers to consider the private sector in their tendering process,^122^ opening up additional opportunities for consultants to earn money in the independent sector (also referred to as revised consultant contracts). As a result, in 1984, 85% of consultants engaged in some private practice – the highest figure since the NHS’s founding.^123^ Between 1979 and 1985, the number of private-sector beds increased from about 6,500 to 10,200, with for-profit hospitals accounting for half of the total.^124^ However, the government’s attempts to commission for-profit clinics to reduce NHS waiting lists proved unsuccessful.^125^ One of the problems was that the marginal costs of using private facilities were higher on average.^125^ These higher costs reflected: (*a*) very high private physician rates (according to Laing, up to 5 times higher than in other countries^126^) and (*b*) scale disadvantages because many of the private clinics were very small.^12^ Private providers were able to demand high prices from private health insurers because of limited competition in the private sector and because patients perceived private care as a luxury product.^127^

#### The Internal Market and the Purchaser-Provider Split

In 1991, local health authorities were given the responsibility of commissioning hospital care (under the so-called purchaser-provider split) and were allowed to purchase services from private for-profits under certain circumstances. Many NHS trusts reformed their pay beds into Private Patient Units in separate complexes that mimicked the more luxurious surroundings of the private sector. The private sector perceived this development as a threat to its business and argued that it constituted unfair competition.^128^ While the purchaser-provider split did not substantially change the NHS provider markets – with public providers continuing to enjoy local monopolies and encounter little competition – the outdated capital (i.e., buildings and equipment) infrastructure of the NHS and increasing waiting lists nourished the continuing growth of the for-profit hospital industry.

In the late 1990s, Tony Blair’s New Labour Government initiated massive investments in the NHS. Consultants were offered substantial pay raises if they agreed to work more NHS-hours.^129^ By 2012, the proportion of consultants engaged in private practice had fallen to 53%, down from approximately 70% in 1993.^130–132^ NHS consultants were also discouraged from relying on private earnings by the imposition of the “10% rule,” which forbade those on full-time contracts from earning more than 10% of their income from private practice.^131^ Gradually, the NHS became more appealing to private patients.

These changes also led the for-profit sector to gain interest in selling services to the NHS. In 2002, for-profit independent treatment centers took part in a £1.6 billion program to reduce NHS waiting lists^133^ and in 2005, a second phase was launched with an estimated cost of £4 billion.^134^ Most contracts were given to new foreign providers, which set up special clinics for this purpose. These non-British physicians were typically cheaper to employ and ensured compliance with a prohibition on drawing away NHS staff.^135^ Established private providers observed this new competition with dismay.

The prospects of the new patient-choice policies were also problematic. Under these policies, patients could opt for any private provider willing to accept the NHS’s payment rates. Consequently, private hospital groups increasingly felt pressured to either stay with their existing high-cost business model catering to private patients or to adopt new, low-cost business models for NHS patients. Private insurers also became more critical purchasers, trying to lower costs by stimulating the growth of hospital networks. However, this shift actually favored for-profit groups because of their larger scale and negotiation power. By 2007, the for-profit sector operated almost 75% of all private hospital beds, but overall growth had stalled.^12^

#### The Decade of Austerity

The 2008 financial crisis led to austerity policies that had a negative impact on private care, as illustrated by the negative profit margins of BMI Healthcare, the largest private provider (Table 3). Spire and Ramsay – a global firm that today operates 480 hospitals worldwide, including in the United Kingdom – fared better over the long term (Table 3). These woes were largely attributable to the decline of private insurance, with enrollment falling steeply in the past decade.^51^ Private hospitals were only partly able to compensate for this decline by increasing services covered by low-margin public funding and a small number of self-pay patients.

**Table 3.** Profit Margins of the Largest U.K. Hospital Chains.

	**2010**	**2011**	**2012**	**2013**	**2014**	**2015**	**2016**	**2017**	**2018**
BMI Healthcare^a^	−3.5%	−6.3%	−8.5%	−2.4%	−1.7%	0.0%	−10.0%	−4.2%	1.9%
Ramsay^b^					9.0%	9.7%	10.4%	10.4%	6.0%
Spire Healthcare^c^				−6.8%	−0.8%	8.3%	7.9%	2.4%	0.9%

Sources: Bureau van Dijk,^96^ Ramsay,^136–138^ and Spire healthcare.^139–141^

^a^BMI Healthcare figures are based on Earnings Before Interest and Taxes (EBIT).

^b^Figures are based on EBITDA of Ramsay’s hospitals in the United Kingdom.

^c^Figures are based on EBITDA.

The Conservative government’s stringent austerity policies held health care expenditures flat over a 4-year period (2011–2012 to 2014–2015) while the government opened opportunities for private providers to deliver services paid for by the NHS. The white paper *Equity and Excellence: Liberating the NHS* mandated that patients be allowed to seek care from any provider of their choosing and that quality guidelines and prices be harmonized.^142^ The Health and Social Act (2012) introduced Commissioning Groups – one of the most far-reaching pieces of legislation in the history of the NHS.^143^ Private providers were finally granted the right to bid for contracts to deliver NHS services and won one-third of all contracts (although 85% of the funds were still awarded to NHS providers).^144^ With this increased access to NHS contracts, the private sector now derives 32% of its revenues from lower-margin public funding, up from 5% a decade ago.^51^

Austerity measures also affected NHS consultants. Because of a pay freeze put in place in 2010 that applied to all NHS staff, total gross earnings fell by 2.6% between 2009 and 2015, and junior doctors and consultants alike had to tighten their belts.^145^ Moreover, the private sector’s financial problems curtailed consultants’ opportunities to supplement their incomes.

#### Brexit and the Future of For-Profit Hospitals in the United Kingdom

Although for-profit providers can now compete for NHS resources, popular suspicion about a post-Brexit “privatization of the NHS” persists. In addition, the BMA has become more critical of the private sector and highlights the risks associated with contracting private hospitals to deliver NHS care.^146^ They and others have voiced concern about the lack of transparency of private hospitals.^146,147^

The question at present is what impact the Long-Term Plan for the NHS and the COVID-19 crisis will have on the private sector. The Long-Term Plan delegates greater autonomy to the United Kingdom’s new leading integrated care systems – its version of ACOs^148^ – to manage services. These systems may enjoy even greater latitude to contract out services to private partners. At the time of this writing, the COVID-19 outbreak hit the United Kingdom (especially England) hard in terms of excess mortality compared with other continental European countries.^149^ The huge backlog in maintenance of NHS buildings – estimated to total £6.5 billion^150^ – and the strain on the public budget caused by the medical catastrophe and impending recession may push the government to seek further support from the private sector. During the COVID-19 outbreak in spring 2020, the government has block-bought the private hospital capacity.^111^

### Germany: Privatization of the Public Sector

In the early 20th century, affluent families usually received inpatient and outpatient hospital care at proprietary clinics. From 1931 onward, hospitals were required to focus only on inpatient treatment, and most of their physicians were salaried.^151^ However, in rural areas, due to shortages of local ambulatory specialist care, some proprietary staff hospitals continued to function as “open staff” facilities in which a combination of outpatient and inpatient care were still permitted.^12^

#### Short on Money After World War II

World War II destroyed the German hospital sector. West Germany became a federal republic, with powers vested in the states if not explicitly granted to the federal government. In health care, many powers were delegated to nongovernmental bodies, with self-regulation (including the allowance of mixed hospital ownership) serving as a guiding principle. Thus, although for-profit providers and their participation in health care delivery were legally uncontroversial, the for-profit hospital sector’s market share declined until German reunification in 1989.^12^

After World War II, the hospital sector was in a dire state and had to be completely rebuilt. However, capital was scarce, and public needs other than hospitals were prioritized. Hospitals incurred significant deficits annually,^152^ which, in many cases, had to be covered by the states and the municipalities that owned them. The federal government and sickness funds that paid the hospitals focused on keeping contribution rates low. As a result, policies during the 1950s and 1960s largely prioritized public and nonprofit hospitals over for-profit hospitals.^152^For-profits could therefore not fall back on deficit funding from local governments, capital subsidies from the states, or endowments and free labor from the voluntary sector. Two niche markets survived: (*a*) one that offered profitable services and better amenities to well-off, privately insured patients whose insurers paid rates 1.5 to 2 times as high as those paid by sickness funds^153^ and (*b*) one that provided access to inpatient facilities for ambulatory medical specialists in sparsely populated rural areas, especially Bavaria.^154^ Nevertheless, by 1969 the proprietary hospitals’ share of acute care beds had fallen to 4.3%, down from almost 8% in the late 1950s.^12^

#### Dual Funding: Capital Versus Current Costs

The pressing financial situation of the hospital sector was finally addressed in 1972. The Hospital Finance Act (HFA) (which required a change in the constitution) initiated systematic planning of hospital infrastructure, with the federal government assuming responsibility to co-fund hospital investments. The HFA introduced dual funding, whereby the states (Länder) and federal government were jointly responsible for funding capital investments. The amounts invested were based on state planning and calculations of operating costs by sickness funds.

While the HFA greatly augmented hospital funding, it prevented for-profit hospitals from receiving capital subsidies for about the first 10 years of its existence. These entities were excluded from hospital planning. Moreover, private physicians in for-profits were not permitted to charge sickness funds higher rates for their services than other providers. Sickness funds could only contract with for-profit hospitals under limited conditions and were not required to contract with physicians who were not listed in state hospital plans.^155,156^ Thus, for-profit hospitals either had to operate with a lower cost base than their peers or had to rely on private patients.

Most states were unable to meet demands for public capital and soon shortages became evident, the so-called *Investitionsstau.* Additionally, in 1984 – a year after a right-leaning party came into power – the federal government stopped contributing to hospital capital investment and reduced hospital investment budgets.^157^ At this point, rules were changed to permit states to incorporate for-profit providers in their hospital plans.^158^ Additionally, hospital operating payment plans increasingly included funding for small investments. Many municipalities struggling to support heavily indebted public hospitals debated privatizing them; in 1984, the city of Hürth, unwilling to continue meeting its hospital’s annual deficit, was the first to turn to privatization.^159^

#### Reunification and the Boom in For-Profit Hospital Care

In 1989, German reunification triggered a for-profit hospital boom. Reunified Germany had to cope with large numbers of neglected public hospitals in the eastern part of the country and privatization seemed an appealing solution. For-profit hospitals were according prominent roles in most of the new states.^157^ Corporate tax reductions also improved the investment climate.^47^

**Figure 3.**
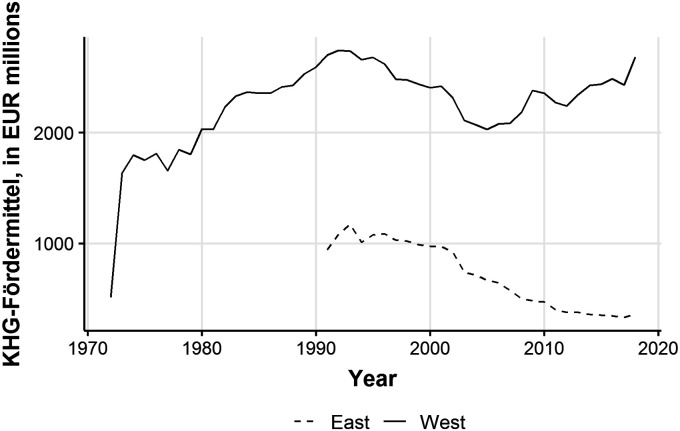
Total annual hospital capital funding (Krankenhausfinanzierungsgesetz) 1970–2020. *Source:* Arbeitsgemeinschaft der Obersten Landesgesundheitsbehörden.^160^

In 1989, Rhön-Klinikum was the first hospital group to be listed on the public stock exchange. Other hospital chains soon emerged, including Fresenius/Helios and Asklepios. Such publicly traded hospital groups were well-positioned to take over and consolidate struggling hospitals in Eastern Germany. They paid very low (or no) acquisition costs, while taxpayers were providing relatively generous capital funding (Figure 3). By 2001, the privatization of hospitals to for-profit status was 22% in Thuringia, 20% in Saxony, 16% in Mecklenburg, 12% in Berlin, and 11% in Brandenburg, with only Saxony-Anhalt lagging somewhat behind.^12^

The financial situation of the hospitals in West Germany stagnated, partly because huge state investments were being made to improve living standards in East Germany (e.g., infrastructure investments).^161^ This eventually triggered privatization in the West as well. For-profit hospital market share in Hesse and Schleswig-Holstein grew to over 20%. However, in densely populated North Rhine-Westphalia, which had many private nonprofit hospitals, for-profit market share remained under 5% in 2007.^12^

Physicians were generally amenable to for-profit hospital conversions, in part because they typically offered more favorable terms of employment.^162^ (However, since 2008, physicians in public hospitals have received larger salary increases).^163^Public-sector wages today are uniform across hospitals,^164^ while labor agreements set private-sector wage scales that vary from hospital to hospital.^165^ At present, physician pay is generally lower in for-profit hospitals (Table 4), although Helios is an exception.^166^ The income of physician executives in for-profit hospitals, however, is often tied to the financial performance of the hospital and in some cases may be significantly higher than the amounts called for in the labor agreements.^167^

**Table 4.** Monthly Gross Pay Scales (in Euros) for Medical Specialists According to Number of Years of Service, 2019.

	1–3 years	4–6 years	7–8 years	9–10 years	11–12 years	13 years
Public hospitals	5,956	6,455	6,894	7,149	7,399	7,649
Rhön Klinikum	5,579	6,040	6,514	6,745	7,031	7,188
Asklepios	6,025	6,530	6,965	7,230	7,475	7,625
Helios^a^	6,123–6,305	6,634–6,938	7,182–7,486	7,546–7,608	7,729–7,791	7,791

Sources: Vereinigung der kommunalen Arbeitgeberverbände, Helios, Asklepios, Rhön Klinikum.^168–171^

^a^Helios is the only one with where pay scales rise with each year of experience rather than every 2 to 3 years, hence the range in the cells.

Hospital payment reforms introduced in the 1992 Health Care Structure Act and the 1997 Hospital Restructuring Act gradually weakened the dual funding structure and paved the way toward a DRG-like prospective payment system. Although the principle of the dual funding structure remained intact, these acts introduced fixed budgets and spending caps to curb costs. In other words, these reforms put the hospital sector under financial pressure. Whereas between 1988 and 1992, state subsidies covered almost all capital investments, between 1993 and 1997 this fell, with almost 40% of hospital capital investments coming from sources other than state subsidies.^172^ As for-profit hospitals received lower levels of state capital subsidies than public hospitals, they were less affected and, therefore, gained a certain comparative advantage.^157,173,174^

#### Merkel’s Legacy on For-Profit Hospital Growth in Germany

Angela Merkel’s chancellorship has produced no major reforms in the health care sector,^175^ but incremental policy changes during her tenure may have had profound long-term effects. First, the 2015 Health Care Strengthening Act, which aimed to foster integration among providers and to integrate care models, weakened the separation between inpatient and outpatient care and allowed hospitals to provide some ambulatory care.^176,177^ This legislative change opened up a new market for the for-profit sector. Second, the 2016 Hospital Structure Reform Act called for quality-based hospital planning and pay-for-performance plans and aspired to reduce capacity, consolidate care into fewer facilities, and control inpatient utilization. As a result, the Fixkostendegressionsabschlag (FDA) now fines hospitals that increase the volume of care they deliver. Some predict that this legislation may incentivize hospitals to provide more lucrative services and avoid provision of less profitable ones.^178^

The hospital sector has prospered under Merkel’s regime, with the profit margins of all hospitals rising by approximately 1 to 3 percentage points (authors’ own calculations).^178–180^ Yet, the profit margin of the for-profit sector as a whole remains significantly higher than that of the other ownership types.^178–180^ Profit margins of the largest for-profit hospitals chains depict a similar pattern, with relatively stable profit margins over the years (Table 5).

**Table 5.** Profit Margins Largest Chains in Germany.^a^

	2010	2011	2012	2013	2014	2015	2016	2017	2018
Asklepios Kliniken	5.7%	5.3%	6.2%	5.3%	6.7%	6.6%	7.7%	8.6%	
Helios Kliniken (Krefeld, Schwerin, Duisburg, Hildesheim, München)	5.8%	3.6%	4.0%	3.5%	−6.6%	8.4%	11.1%	9.6%	11.6%
Sana Kliniken AG	3.7%	3.7%	3.1%	3.2%	3.7%	4.0%	4.9%		4.7%

Source: Bureau van Dijk.^96^

^a^Figures are based on EBIT. Helios is the biggest German chain, but figures only reflect the profit margins of the 5 mentioned hospitals between brackets.

At present, Germany has a large and prosperous for-profit hospital sector, and the financial environment remains favorable for for-profit hospitals. However, the competition authority has recently raised concerns about the high level of concentration in the private hospital market,^181^ making it more difficult for for-profit chains to continue to expand domestically. Partly for this reason, Fresenius – the largest German hospital firm, operating under the hospital brand name Helios – took over Quirónsalud to expand in Spain and thus become the largest hospital chain in Europe.

### The Netherlands: A Counterfactual Case to For-Profit Hospital Growth

#### Why the For-Profit Hospital Industry Did Not Kick Off in the Netherlands

Dutch for-profit hospitals have never flourished. Nonprofit hospitals have had a strong foothold in the health care system since the 1850s because of the reliance in Dutch society on religious communities (rather than government) to provide social services – so-called pillarization. For a long time, nonprofit hospitals were also open staff, which discouraged physicians from building their own, competing facilities. Thus, the drivers of proprietary hospitals in the United States, the United Kingdom and Germany (lack of physician access and lack of amenities and services for the well-off) were not prominent in the Netherlands, and nonprofit hospitals gradually became the dominant providers.

After World War II, hospitals wanting to make new capital investments were required to obtain a certificate of need from the local municipality, but the local government bore no responsibility for funding the investment. Instead, the social insurance plan was required to include reimbursement for approved capital expenditures (but not return on equity) in each hospital’s per-diem rates, making hospital capital investments virtually risk-free and obviating the need for hospitals to accumulate cash for down payments.^12^ As a result, a construction boom followed, but with little demand for private capital to fund hospital investments^182^ and little profit incentive for investors,^183^ conditions were not favorable for the growth of for-profit hospitals.

#### Legal Prohibition of For-Profit Hospital Ownership

The 1971 Hospital Facilities Act (HFA) was a response to the burst of construction and fears that costs would escalate. The act centralized hospital planning (by removing municipalities’ right to approve new hospital investments) and provided a mechanism to enforce cost-containment policies. The HFA also prohibited for-profit hospitals from receiving certificates of need or reimbursements from the social insurance plan.^184^ This legal restriction was the final door to close on the prospects of for-profits (although theoretically, it remained possible for for-profits to purchase existing nonprofit hospitals and offer services to privately insured patients). Private insurers, which covered the wealthiest 30% of the market, were strongly embedded in the corporatist decision-making structures of Dutch health care and, unlike in the United Kingdom and Germany, did not push for the development of private hospitals.

#### Managed Competition, But Without For-Profit Hospitals

Managed competition theory profoundly influenced Dutch health policy. The 2006 Health Insurance Act was the flagship effort to create an entirely private health care system, based on the principles of regulated competition, with hospitals paid through DRGs.

Under the reform, private insurers could compete for customers – although they were prohibited from distributing profits to owners or shareholders – and were given increasing latitude to negotiate prices with providers; in 2012, prices for 70% of inpatient DRGs were subject to negotiation.^185^ With managed competition being the new policy paradigm, for-profit hospital ownership was seen by many, including the High Court,^184^ as the logical next step.^186^ Moreover, the 2005 Health Care Institutions Admission Act, the successor to the HFA, had simplified regulations and reduced the government’s role in hospital planning, which seemingly opened the way to lift the ban on for-profit hospitals. Indeed, the government stated that it was prepared to lift the ban by 2012 and that hospitals would be permitted to become private companies as long as they did not pay any dividends to investors until the ban was formally lifted.^184^ Twelve hospitals converted to private ownership status, although not all sought to become for-profits (authors’ calculations using annual reports). In 2008, the remaining certificate of need regulations and capital reimbursement plans were phased out. Hospitals were then free to (re)develop property. However, under prospective payments, they became exposed to investment risks.^187^

In anticipation of the lifting of the ban on hospitals operating in a for-profit mode, private investors acquired 3 hospitals: MC (medical center) Slotervaart, MC Ijsselmeerziekenhuizen, and Red Cross Hospital. In the case of the Ijsselmeerziekenhuizen, the government donated approximately €20 million to save it (2008).^188^ The 2 MC hospitals eventually ran into severe financial problems and by late 2018 were bankrupt; MC Slotervaart had to close its doors permanently in 2019, and the other hospital was merged with a local nonprofit.^189^ An independent committee investigating the causes of the bankruptcy cited, among other factors, the medical staff’s suspicion that shareholders extracted money from the hospital through rent paid to an affiliated real estate firm. These suspicions fueled a toxic relationship between the medical staff and the shareholders/board of directors and made it difficult to reorganize the hospital.^190^ The Red Cross hospital remains in a stronger financial position^191^ and is currently the only surviving investor-owned hospital.

#### Is There a Future for For-Profit Hospitals in the Netherlands*?*

The government’s promise to lift the ban on for-profit hospitals’ distribution of dividends was always controversial, and left-leaning parties that opposed lifting the ban were sometimes supported by the Christian Democrats. In 2013, the House of Representatives approved a law that was favorable toward for-profit hospitals, but that still imposed several restrictions: for example, no profits could be distributed for the first 3 years, hospitals would have to maintain solvency ratios of at least 20%, and the hospital would have to receive a positive rating from the Health Care Inspectorate.^192^ However, in 2014, the Dutch Minister of Health, Edith Schippers, asked the Senate to delay voting on the law,^193^ claiming it was not ready for implementation. Political considerations apparently contributed to the postponement; it has since come to light that the Senate would probably have voted against the law.^194^

In 2017, the newly formed government promised to make a decision in 2018 on whether to proceed with the law, but subsequently again postponed this to 2019.^195^ In October 2019, the Minister of Health encountered political obstacles because of the widely publicized defaults of the commercially owned hospitals, described above, and scandals regarding excessive profits in the home care sector.^196^ This was the straw that broke the camel’s back; the government announced that it was taking the repeal of the ban on for-profit hospitals off the table.^197^

For the foreseeable future, the Dutch hospital sector will remain exclusively private, not-for-profit. However, it is notable that the nonprofit hospitals have greatly improved their capitalization. Solvency ratios (assets/liabilities), which in 2002 were estimated to be 7%,^198^ now average nearly 25% – an increase that has occurred mostly since the 2006 market reforms (Table 6). Because owners/managers of other types of health care providers have developed creative accounting tricks to circumvent the ban on distributing profits,^199^ such high levels of solvency might well draw the attention of investors in the future.

**Table 6.** Solvency Rates for Dutch Hospitals (2007–2017).^a^

	**2007**	**2008**	**2009**	**2010**	**2011**	**2012**	**2013**	**2014**	**2015**	**2016**	**2017**
Average solvency rates	11.9%	12.7%	14.1%	14.8%	16.8%	18.9%	20.8%	22.4%	20.9%	21.6%	23.7%
Median solvency rates	12.1%	12.6%	12.4%	13.8%	18.1%	19.6%	20.5%	22.5%	21.2%	22.4%	24.4%

Source: CIBG.^54^

^a^Authors’ calculations.

## Discussion

### Why the For-Profit Hospital Sector Has Thrived in Some Countries, but Not Others

After a period of decline during the first part of the 20th century, the for-profit hospital sector has grown rapidly in some, but not all, developed nations in recent decades. For-profit hospital market share rose steeply in Germany after reunification and somewhat less briskly in the United States since the 1960s. However, growth has been slow in the United Kingdom and almost nil in the Netherlands.

In the United States, public Medicare and Medicaid insurance programs implemented in the mid-1960s were favorable toward for-profit hospitals, offering them higher payments than nonprofits. Conversely, the United Kingdom’s NHS sidelined for-profits in 1948, and both the Netherlands (1971) and Germany (1972) excluded for-profit hospitals from most sources of public funding. With the rise of neoliberalism and NPM in recent years, all 4 countries have moved to bolster the role of for-profits, albeit with varying effects.

What explains for-profits’ divergent paths across these 4 countries? Neither our case studies nor previous research suggest that for-profit success is attributable to greater efficiency. Instead, our cross-national comparisons suggest that 3 other factors influence the likelihood of for-profit success (Table 7): (*a*) access to capital funding and reimbursement for services from government health care financing programs, together with the generosity of these reimbursements; (*b*) the extent to which physicians’ financial interests coincide with for-profit interests; and (*c*) the political environment. The first of these factors, the specific, seemingly arcane details of the terms of for-profits’ participation in public health care financing programs – especially access to capital funding – appears most important. Physicians’ ability to realize financial benefit from for-profit hospitals was relevant in the early 20th century, but its importance has since waned. The political environment shapes key health care financing policies, but explicit decisions to ban or encourage for-profit ownership are often short-lived and of lesser importance.

**Table 7.** Assessment of the Impact of Factors That Affect For-Profit Hospitals’ Growth.^a^

		United States	United Kingdom	Germany	The Netherlands
Public funding	Access to funding/reimbursement for capital investments	4	3	4	5
		Stimulated growth	Stimulated growth	Stimulated growth	Prohibited for-profits
	Access to and terms of reimbursement for service delivery from public programs	4	4	4	4
		Stimulated growth	Shaped provision	Stimulated growth	Hindered growth
	Cost-control measures applied to broader hospital sector	4	5	4	3
		Created acquisition targets	Mixed effects	Created acquisition targets	Created acquisition targets
Concordance with physicians’ financial interests	Higher remuneration by for-profit hospitals	4	5	3	1
		Mixed effects	Mixed effects	Mixed effects	Not applicable
Political environment	Supporting for-profit growth	3	3	5	4
		Little debated	Mixed effects	Privatizations encouraged	Vetoed at several points

^a^1: very unimportant, 2: unimportant, 3: neutral, 4: important, 5: very important.

### Public Payment Systems’ Effects on For-Profit Development

Three aspects of public policies regarding provider payments appear important: (*a*) regulations that determine access to capital subsidies and return on investments, (*b*) whether for-profits are allowed to bill public programs for the care they deliver, and (*c*) the effects of system-wide cost-control policies.

After World War II, private funds for hospital investment were scarce in all 4 of the countries we analyzed. Governments stepped in to provide resources to expand hospital capacity through programs that largely or completely excluded for-profits. Unable to access substantial funding to build or modernize facilities, for-profit providers mainly focused on niche markets.

Except for the Netherlands and Germany, for-profits gained greater access to public funding in the 1960s and 1970s. From its inception in 1965 until about 1990, the U.S. Medicare program gave for-profits an explicit competitive advantage in the form of more generous capital payments than were available to nonprofit or public hospitals. Thereafter, the playing field was formally leveled. German for-profits gained formal (but only partial) access to the stream of public health care funds starting in the 1970s and 1980s. However, for-profits’ privileged access to private capital funding through stock sales offered a decisive advantage in the early 1990s and allowed them to take over many East German hospitals badly in need of funds for modernization. In the United Kingdom, the NHS has, since its founding, had a serious shortage of capital funds. Inadequate funding of the public sector created an opening for private providers to attract modest funding from investors. In contrast, the Netherlands banned hospitals from distributing profits to investors, effectively foreclosing the development of for-profit hospitals.

For-profit hospitals in the United States and Germany were granted immediate (United States) or delayed (Germany) access to reimbursement for service delivery from public programs. Conversely, for a long time, the for-profit sector in the United Kingdom relied primarily on private payments, and the sector’s mode of provision – characterized by small-scale clinics offering superior amenities – was shaped by their role, which was limited mostly to providing supplementary services. The recent advent of outsourcing by the NHS has given for-profits access to public payments, although they have struggled to find a profitable business model. The outlier is, again, the Netherlands, where for-profit hospitals were, until 2006 reforms, not allowed to bill the social insurance plan. At present, for-profits may be reimbursed for services, but may not distribute profits to investors.

Several factors contributed to the apparent resilience of for-profit hospitals during periods when cost-containment policies squeeze the hospital sector. For-profits’ ability to tap into private capital when public funding is in short supply may allow them to weather periods of austerity. Additionally, for-profits appear more willing and able to focus on profitable segments of the hospital market (e.g., cardiac and orthopedic surgery in the United States) and avoid unprofitable ones (e.g., care of the uninsured). For-profits are also often particularly skilled at exploiting legal (and occasionally illegal) loopholes in payment policies (e.g., through upcoding). Finally, the enforcement of cost controls may open opportunities for investors to acquire struggling public and nonprofit hospitals at reduced prices, although in the United Kingdom, for-profits’ increasing reliance on NHS funding has left them vulnerable to cuts in public funding.

### Physicians’ Financial Interests and Their Alignment With the For-Profit Hospital Sector

Across all 4 countries, physicians’ financial interests were influential in determining the early development of for-profit hospitals. The United Kingdom – where consultants sought a venue for private practice – was the clearest case. Similarly to the United Kingdom, U.S. for-profit business models depended on attracting (the patients of) self-employed physicians, which led some hospital firms to offer physicians stock or equity arrangements. In Germany, physicians in for-profit (and other) hospitals were generally salaried employees. To this day, nonprofit hospitals in the Netherlands are effectively physician cooperatives that pay specialists – a well-organized group with substantial bargaining power – generous salaries.^11^

In the United Kingdom and Germany, the financial benefits that for-profit hospitals accorded to physicians have somewhat diminished. The number of NHS consultants working in the independent sector in the United Kingdom has declined. In Germany, the wages of physicians in most for-profit hospitals are now lower than those in other hospitals, perhaps reflecting the consolidation of hospital ownership (and hence bargaining power) as a few large chains have come to dominate the market.^200^ In the United States, the number of physician-owned hospitals appears to be declining, and more physicians have become employees of either hospitals or practices owned by venture capital or private equity firms.^201^ Based on our findings, we tentatively conclude that physicians’ roles in stimulating the expansion of the for-profit hospital sector has diminished in recent years.

### Political Decisions and Their (Non-)Influence on For-Profit Market Growth

While political decisions can disrupt and influence the for-profit hospital landscape – particularly through reforms in hospital payment policy – the political color of the ruling party has had surprisingly little impact on the growth of the for-profit sector in the 4 countries we studied (Figure 1). The only explicit effort by left-leaning politicians to roll back for-profit hospital care, during the mid-1970s in the United Kingdom, failed miserably because of strong physician resistance. Instead, these efforts backfired and induced the commercial transformation of the independent sector. In the United States, the advent of Medicare and Medicaid, implemented by a Democratic president as part of a broad expansion of social programs, offered vast public subsidies to for-profit hospitals, accelerating their growth.

On the other hand, policies inspired by neoliberalism and NPM have had mixed effects on for-profit hospitals. In the United States, the turn to market-based policies starting with DRGs in the 1980s has not proven uniquely favorable to for-profits, in part because nonprofit hospitals have increasingly mimicked for-profit strategies. The fall of communism in Germany spurred the privatization of public hospitals in the East, which continued for more than 20 years. In the United Kingdom, the private sector benefited from the NPM ideological shift during Thatcher’s reign. However, despite the neoliberal and NPM-inspired 2006 reform in the Netherlands, for-profit hospitals there have not advanced significantly.

Several factors may underlie the limited effects of political swings on for-profit hospital growth. The hospital sector is inherently rigid: Hospitals cannot be built nor acquire a patient base overnight. Once for-profits have gained substantial market share, their financial power confers political influence that enables them to safeguard their influence. And, relatedly, hospitals, as major employers, often wield strong influence in their local communities, helping hospitals ward off measures that might disrupt their business.

## Conclusions and Policy Implications

Our analysis highlights several factors that influence the size and success of the for-profit hospital sector. The seemingly technical details of how public reimbursement plans treat for-profit providers, particularly regulations related to accessing public capital funding and reimbursement for private capital expenditures, have the greatest impact. Cost-containment measures and payment arrangements, which have squeezed some nonprofit and public hospitals in Germany and the United States, have also stimulated for-profit growth by providing openings for investors to acquire facilities at low costs. For-profit hospitals’ early growth in the United States and in Germany was also abetted by physicians who stood to gain financially. However, the role of physicians in stimulating the expansion of the for-profit hospital sector has apparently waned in recent years, as more power has been ceded to investors. The commercialization of hospital care can be a heated political topic, with left- and right-leaning politicians often holding opposing views. However, the political environment, at least within the spectrum present in the nations we examined, had relatively little direct impact on the growth of the for-profit hospital industry, with the notable exceptions of the United Kingdom in the mid-1970s and Germany in the early 1990s.

### Policy Implications

Decisions regarding public reimbursement plans are critical determinants of the growth of the for-profit hospital sector. Such decisions influence short-term profitability and are often relatively stable and long-lasting. Hence, policy makers seeking to influence the composition of the hospital market should focus on the design of payment plans, particularly the details of capital funding and reimbursement. Intervening to reduce the capital costs for one ownership form relative to others may induce long-run changes in the composition of the hospital sector. Thus, our findings call for closer examination of how capital reimbursement plans “steer” the business of health. Finally, the for-profit hospital sector is quite sticky: Once it has grown, it tends not to shrink. This characteristic is particularly relevant in an era when many hospitals are under financial pressure. Privatizing financially distressed public or nonprofit hospitals is relatively “easy,” but reversing privatization is often strenuous and costly.
